# Restricted health service utilization and subsequent positive self-care behavior during the early COVID-19 pandemic in China

**DOI:** 10.3389/fpubh.2024.1398271

**Published:** 2024-07-09

**Authors:** Zhichao Wang, Zhongliang Zhou, Guanping Liu, Jiao Lu, Xiaohui Zhai, Xiaojing Fan, Sha Lai, Youfa Wang

**Affiliations:** ^1^School of Public Policy and Administration, Xi’an Jiaotong University, Xi’an, China; ^2^School of Public Health, Health Science Center, Xi’an Jiaotong University, Xi’an, China; ^3^School of Public Health, Global Health Institute, Xi’an Jiaotong University Health Science Center, Xi’an, China

**Keywords:** health service utilization, self-care behavior, reasoned action approach, online medical consultation, subgroups analysis, vulnerable population, COVID-19

## Abstract

**Background:**

The reallocation of health resources, epidemic prevention and control measures during the COVID-19 pandemic triggered widespread restricted health service utilization, some residents and patients tried positive self-care behavior to maintain their health, yet the efficacy of this intervention remains unclear.

**Object:**

Based on the reasoned action approach (RAA) theory, this study aimed to investigate the correlation between self-care behavior and restricted health service utilization among adults in China, trying to discover the vulnerable groups and external and intrinsic factors that affect self-care behavior among Chinese adults.

**Methods:**

Data on demographics, socioeconomic, health status, and self-care behavior were collected in “The Early China COVID-19 Survey,” a cross-sectional anonymous online survey of the general population in China. Self-care behavior was measured by four indicators: weight control (WC), physical activity (PA), prevention behavior (PB), and online medical consultation (OMC). The multiple linear models and binary logistic regression were used to examine whether restricted health service utilization (RHSU) is associated with self-care behaviors; also, adjusted multivariate logistic regression was used to analyze subgroup heterogeneity.

**Results:**

In total, 8,428 adult participants completed the survey, the mean OMC score was 1.51 (SD 1.34), the mean PB score was 18.17 (SD 3.44), and the proportion of participants who engaged in WC and PA was 42.30 and 62.57%, respectively. According to the multiple regression model, the RHSU was significantly positively correlated with all four indicators of self-care (WC: OR = 1.34, *p* < 0.001, PA: OR = 1.34, *p* < 0.05, MC: OR = 1.30, *p* < 0.001, PB: coef = 0.16, *p* < 0.05). We also observed some significant differences in the intensity of this relationship by subgroup analysis, precisely, OMC (high vs. moderate vs. low infection-risk level: OR = 1.48; 1.41; 1.19, *p* < 0.1), PA (male vs. female: OR = 1.27;1.06; *p* < 0.05, high vs. Moderate and low infection-risk level: OR = 1.51; 1.17; 1.02, *p* < 0.05), PB (Chronic disease groups vs. no: coef = 0.46; 0.1, *p* < 0.05).

**Conclusion:**

Restricted health service utilization predicts more positive self-care behavior, and the intensity of partial correlation was significantly different in the subgroups of sex, actual infection risk level of the living area, and chronic diseases. These findings highlight the urgent demand for self-care behavior among Chinese adults during the pandemic and provide new insights for developing self-care and reducing the burden on the healthcare system in the long term.

## Introduction

During the first wave of the COVID-19 pandemic, a global lockdown was imposed in response to the rapid spread of the virus; the government’s regulations or restriction measures to curtail virus spread may have an enormous impact on people’s daily lives (1, 2). It is self-evident that the implementation of quarantine measures, lockdown, and social distancing protocols also led to some negative repercussions, which include a significant meltdown in economic growth, an increase in unemployment or financial insecurity, a rise in the cost of living, and a severe impact on health service utilization among the population (3–5).

With the continued containment and control of the COVID-19 pandemic, the general population also experienced unintended potential health risks arising from the restriction of routine healthcare services, specifically, the consequences of the pandemic for the health of these non-COVID-19 patients, including delayed timely detection and treatment, avoided and delayed emergency department visits, unmet healthcare needs and increased rates of exacerbation as resources were reallocated to urgent care for COVID-19 patients (6), researches noted the COVID-19 pandemic has adversely affected the healthcare utilization of the population worldwide, and situation of restricted health service utilization is alarming (7, 8). A national longitudinal study from China found the most considerable negative impact of the COVID-19 pandemic on health services utilization was observed between Jan 2020 and Apr 2020, with approximately a 32% reduction in hospitals, a 22% reduction in community health centers, and 27% reduction in township centers (9). Studies from Europe have claimed that during the COVID-19 pandemic, individuals with acute myocardial infarction, stroke, heart failure, and other chronic cardiovascular diseases experienced a significant reduction (40%) in hospitalization rates and emergency department visits compared to baseline (10). Globally, a large study indicated that global healthcare utilization showed a median decline of 37.2% between the pandemic and pre-pandemic periods, ranging from 19.8 to 50.5% (8). In short, the fear of infection and reduced availability of medical services have driven down non-COVID-19 healthcare utilization. The demand of the general population, particularly vulnerable groups like chronic patients, to improve their health status is a difficult task under normal circumstances, the obvious barriers imposed by the COVID-19 pandemic have considerably increased this difficulty. Meanwhile, considering the healthcare system supply is limited, it appears to be a moral dilemma between allocating medical resources to contain the spread of the virus or providing adequate healthcare service during the pandemic. Nevertheless, it also indirectly drives us to be more concerned about exploring strategies and measures to address the restricted health service utilization more effectively.

## Background

Since the outbreak of the COVID-19 pandemic, the infection spread has come in waves in China, restrictions on health services were occasional (9), and growing research has focused on self-care behavior and recommended it as a response strategy to tackle the potential health harms arising from restricted health service utilization (11, 12). Drawing on the various relevant literature, the restricted health service utilization (RHSU) not only hinders surveillance of health status but also will likely lead to some worse outcomes if it continues, such as unhealthy lifestyles, treatment interruption, and worsening of chronic disease symptoms (13), all of which could lead to serious health problems. To alleviate this potential health crisis, the people with intentions to maintain health and those with chronic diseases preferred to adopt non-therapeutic measures, including physical activities, dietary habits, self-care monitoring and online medical consultation after the outbreak (14–16). Previous studies found that several types of health service utilization restrictions were associated with self-care activities, such as both primary and specialized care (17), personal care aides (18), primary mental health care service (19) and chronic disease treatment (20, 21). Drawing upon evidence from related research, the RHSU might directly or indirectly motivate the general population to compromise in daily choices like consumption habits, diet, exercise, self-care activity and medical consultation, and most people had mitigated potential health harms through these behavioral changes (14–16, 22). For instance, a study of stroke survivors stated that self-care behavior such as physical activity, diet, weight control, smoking cessation, and abstinence from alcohol could help those with restricted health services to reduce systolic and diastolic blood pressure and decrease the occurrence of complications such as hyperglycemia and diabetes and keep disease stable (23). Medical practitioners have also suggested that adopting self-care activities and improved self-care behaviors may play a critical role in maintaining health or preventing immediate and subsequent complications during the COVID-19 pandemic (24).

Specifically, self-care behavior is recognized as an essential and valuable behavior because it emphasizes the positive role of people in maintaining their health, and common individual self-care activities include engaging to improve health status, prevent disease, limiting illness, and regain health (25), the beneficial effects of self-care include improved well-being and lower morbidity, mortality, and healthcare costs (21). Previous research has revealed the complexity of self-care and illustrated the wide variety of factors that influence the decisions individuals make about engaging in self-care (13–16); it also investigated the difficulty performing self-care among special populations (e.g., multiple chronic conditions, severe mental illness, low health literacy) (26); and sustained evolving technology to enable individuals to manage their conditions and improve the efficiency of self-care (27), the multiple disciplines are actively studying self-care and contributing variable knowledge to the topic nowadays (28). However, the current literature suggests that self-care is still under-appreciated and insufficiently understood; there are many challenges to the prevalence of self-care behavior (26). Thus, there is a need to explore the mechanisms underlying self-care behavior further to drive relevant policy development, especially for those countries and regions with a growing burden on health service systems.

### Research gaps and the present study

Previous studies have been conducted on self-care behavior in the older adult, chronic disease patients, non-communicable disease patients, and healthcare professionals (14–16, 28). However, there is still a lack of continuity across research initiatives, and few studies have investigated the changes in self-care behavior among whole adult populations during the pandemic. In addition, some research noted that demographic, socioeconomic status, and health status determine the acceptance and engagement of self-care behavior interventions (26, 29). However, the direct evidence for the relationship between the RHSU and self-care behavior is limited and controversial, and the discussion about the possible differential impacts of RHSU on self-care behavior of individuals with varying health status may differ is also not well substantiated. These knowledge gaps may lead to a deficiency in the comprehensive understanding of the underlying mechanisms of self-care behavior in the current Chinese population and diminish the identification of vulnerable groups with worse self-care behavior. In addition, although some studies have supported the viability of self-care behavior as a strategy to address the RHSU problem and reduce the burden on the healthcare systems, the direct evidences are limited and controversial.

Based on the literature review, we found that the instruments used to measure self-care behavior consisted of several main aspects, such as health consciousness, nutrition, physical activity, sleep quality, medical management of the disease, seeking social support, and adherence to the recommended regimen (30–32), however, previous studies have commonly used single or one-dimensional indicators to measure self-care behavior. Therefore, we decided to establish indicators to measure self-care behavior across two main dimensions, including autonomous behavior and consultative behavior (33). Autonomous behaviors are implemented directly by the patient, such as changing activity or taking weight control to make the symptoms decrease or go away and adjusting health habits to avoid infections. Consulting behaviors are based on guidance from healthcare providers; for example, call your healthcare provider for advice and seek health consultation by using other channels. Additionally, the COVID-19 pandemic provides a particular perspective on whether self-care behavior could be considered an effective strategy for mitigating the health risks caused by restricted health service utilization. Since the reason for the impact of this restricted health service utilization is mainly exogenous and individuals receiving routine medical care or medical consultations also experienced health risk shocks caused by the COVID-19 pandemic. Furthermore, previous research on the relationship between health service utilization and health behavior has occasionally been controversial (18, 20); for example, when experiencing restricted health service utilization, some residents and patients may prefer to receive health service elsewhere rather than engage in more positive self-care behavior. However, the COVID-19 pandemic significantly affected nationwide health service utilization (9), which indirectly helps us to rule out some confounding factors.

According to the reasoned action approach (RAA), a common theory used to understand health behaviors (34), we assume that every individual has the willingness to maintain health and confidence to control their health behavior, also fully aware of the consequences of RHSU and the norms of self-care behavior, then we could assume RHSU is targeted for interventions to change self-care behavior. In our study, we expect that adults who experienced RHSU would be more likely to report positive self-care behavior to maintain their health. However, more evidence is required to explore differences in risk perceptions and self-care behavior at the individual level.

Overall, in this study, we aimed to explore the following question based on a large nationally representative sample:(1) the relation between restricted health service utilization (RHSU) and self-care behavior among Chinese adults during the COVID-19 pandemic; (2) whether the relationship between self-care behavior and restricted health service utilization (RHSU) was different across social groups (i.e., male, female, 18–44 age, 45 and above age; chronic diseases adults, no-chronic diseases adults; adults living in the low, moderate and high infection-risk level area).

## Methods

### Sample collection

Data on demographics, socioeconomic, health status and self-care behavior were obtained from an anonymous online survey called the “2020 China COVID-19 Survey,” which was collected between late April and mid-May 2020. It was collected via WeChat, which is a popular social media tool that has become an essential part of the daily work and life of Chinese adults. The primary aim of the 2020 China COVID-19 Survey study is to explore whether health disparities by age, sex, race, living condition, or socioeconomic status emerge or worsen throughout the pandemic; this survey has been used in other articles in China during the COVID-19 pandemic (35, 36). This structured questionnaire compasses seven topics:

1. The demographic and socioeconomic characteristics.
2. Health status, including chronic diseases during the COVID-19 pandemic.
3. Awareness, attitude, knowledge, and practices toward COVID-19.
4. COVID-19 experiences and impacts.
5. Medical consultation habits.
6. Behaviors that could prevent the spread of COVID-19.
7. Lifestyle habits.

To ensure data accuracy and integrity, one project manager was recruited in each province to coordinate the province-wide survey and organize survey training, and six to twelve local investigators were recruited in each city based on household incomes to distribute online questionnaires and control the survey quality. After being trained in online data collection, each local investigator was asked to send the online questionnaire directly to 20–30 local households in their social networks, including relatives, neighbors, friends, and workmates. Each eligible participant was invited to complete the online questionnaire, which they completed in an average of 8.5 min. Participants are given an appropriate gift when they complete the questionnaire. Some of the older adult could not participate in the online survey due to their age and education level; regarding this group, we decided that relatives living with them obtain their answers through oral questioning and fill out the survey based on their options (26). When this survey began, the COVID-19 pandemic had already caused more than 83,000 infections in mainland China and over 4,600 deaths (37). The pandemic was generally disseminated, with clusters of outbreaks caused by transmitted cases occurring in some areas. Since April 2020, the corresponding preventive and control measures in most provinces in China have been downgraded from emergency response to regular management, with social quarantines, blockades, and travel restrictions identified and implemented according to regional risk classifications. In this study, we used targeted stratified convenience sampling to select residents in China’s eastern, central, and western regions. Our survey included 8,428 adults aged 18 years and over 31 provinces in China. The survey was completed voluntarily and anonymously, and because of the high standardized quality control of the questionnaires, the baseline survey response rate is good. All subjects gave informed consent before participating in the survey, and the protocol was approved by the Ethics Committee Committee of Xi’an Jiaotong University (No. 2020–1,172).

### Outcome variables

In our study, we used four indices, including weight control, physical activity, prevention behavior, and online medical consultation, to measure self-care behavior during the COVID-19 pandemic as the outcome variable in our analyses. Specifically, Weight control and physical activity are two typical variables in autonomous behavior dimensions; we dichotomized both variables to indicate if participants engaged in weight control or physical activity (Yes/No) during the COVID-19 pandemic. Prevention behavior was assessed using a scale consisting of four items derived from some questionnaires widely used to assess the disease control and prevention of populations during the outbreak of COVID-19 (3, 38, 39); the participants were asked about how often they had practiced the following preventive action: (1) Wear face mask in public settings, (2) Wash your hands after a trip outside, (3) Avoid unnecessary outings as much as possible, (4) avoid gathering as much as possible. Each item was rated on a 5-point Likert scale, and response options included: Never = 1, Rarely = 2, Occasionally = 3, Sometimes = 4, All the time = 5. Total scores ranged from 4 to 20, and higher scores mean better Prevention behavior. The Cronbach’s alpha of this scale was 0.949. Regarding online medical consultation, participants were asked if they had used the following approaches to seek medical consultation during the COVID-19 pandemic; option approaches include online consultation, video consultation, telephone consultation service, and mail-order or personal delivery pharmacy. In this case, each option counts for 1 point, and the total score ranges from 0 to 4. Higher scores indicate that participants had used more approaches to seek medical consultation, and Cronbach’s alpha of this scale was 0.782. To differentiate medical consultation capacity from individuals, based on the average of participants’ response scores, we defined participants who chose two or more of the four options as “above-average performance in medical consultations.” Thus, Online Medical behavior can be regarded as a binary variable (No = below average, Yes = above average).

### Independent variable

RHSU was the independent variable, measured by recording whether the participant’s routine medical care or medical consultations were restricted due to the COVID-19 pandemic. Hence, it was a binary variable (No = unrestricted due to COVID-19; Yes = restricted due to COVID-19).

### Control variables

The control variable in our model includes demographic and socioeconomic characteristics mainly including region (city/rural or town), sex (male /female), age (18–44/45 years or above), education level (obtaining a bachelor’s degree/no bachelor’s degree), marital status (married/unmarried or divorced or widowed), the household income gradient in the last year (i.e., before the pandemic) was divided into three tertiles (low = 1st tertile/middle = 2nd tertile/ high = 3rd tertile), health conditions included chronic medical conditions (yes/no), self-rated health status (fair or poor/good/very good) and actual COVID-19 infection risk level (low/moderate/high) in the participant’s place of residence. In addition, this study collected six variables on individual perceptions and impacts during the covid-19 pandemic, including whether participants think one of their family members had been infected with COVID-19 (yes/no), whether they had experienced food or medicine shortages (yes/no), whether they or their family members lose their job due to COVID-19 (yes/no), the degree of difficulty your family experiences in daily activities caused by COVID-19 related financial strain (no difficulty at all /mild difficulties/extreme difficulties), and the degree of how serious of a public health threat they think COVID-19 is or might become (low/midden/high).

### Statistical analysis

From the survey, we collated the required descriptive statistics, including frequencies (*N*) and percentages (%) or means (M) and standard deviation (SD) and their 95% confidence intervals (95%CI). Then, we use multiple linear models and binary logistic regression to examine whether RHSU was associated with self-care behavior outcomes. In the process, we measured self-care behavior through four indicators and introduced the following control variable into the model for each indicator: demographic and socioeconomic variables (age, sex, marital status, educational level, residential area, and household income level in the last year), health condition (chronic disease, self-rated health) and COVID-19 related variables (lost job due to COVID-19, food shortage, experienced COVID-19 infection, drug shortage, perceived risk of infection, the degree of difficulty in daily household activities and infection risk level of living area), which were chosen based on the knowledge of the available literature related to the topics (11, 12, 22, 36). Finally, the adjusted multivariate logistic regression was introduced to analyze subgroup heterogeneity in sex, age, chronic disease, and actual infection risk level of the respondent’s residence; the differences in self-care behavior outcomes between subgroups were tested using the Chow test (40). Statistical tests were considered significant if *p* < 0.1. The association between participant characteristics and study outcomes was quantified using standardized regression coefficients (β) and odds ratios (ORs) and their 95% CIs. All statistical analyses were performed using STATA statistical software version 17.0 (StataCorp et al. Station 77,845, USA). *p* < 0.05 (two-sided) was considered statistically significant.

## Results

### Basic characteristics

Table 1 summarizes the characteristics of the sample of participants who completed “The 2020 China COVID-19 Survey” and illustrates whether participants’ routine healthcare or medical consultation has been restricted due to COVID-19. A total of 8,428 participants were included; the average age was 32 years (SD 9.95 years, range 18–79 years), and 19.97% (*N* = 1,683) of them suffered from at least one chronic disease. In addition, 3,978 (47.2%) participants’ routine medical care or medical consultations were restricted by COVID-19, and 9.31% (*N* = 785) of their family members got infected with COVID-19. Most of the differences in demographic and socioeconomic characteristics, health status, and COVID-19-related variables among the two RHSU subgroups were statistically significant.

**Table 1:** General characteristics of participants [*N* (%)].

Variables		Total (*N* = 8,428)	Restricted health service utilization		*P* value
			No (*N* = 4,450)	Yes (*N* = 3,978)	
Region	City	5,085 (60.33)	2,516 (56.54)	2,569 (64.58)	< 0.001
	Rural	1,276 (15.14)	685 (15.39)	591 (14.86)	
	Town	2067 (24.53)	1,249 (28.07)	818 (20.56)	
Actual infection risk level of living area	Low	3,787 (44.93)	2,310 (51.91)	1,477 (37.13)	< 0.001
	Moderate	3,065 (36.37)	1,627 (36.56)	1,438 (36.15)	
	High	1,576 (18.70)	513 (11.53)	1,063 (26.72)	
Married status	Unmarried/divorced/widowed	2,952 (35.02)	1,477 (49.97)	1,477 (50.03)	< 0.001
	Married	5,476 (64.97)	2,978 (54.33)	2,501 (45.67)	
Sex	Male	3,694 (43.83)	1761 (39.57)	1933 (48.59)	< 0.001
	Female	4,734 (56.17)	2,689 (60.43)	2045 (51.41)	
Age (years)	18–44	7,387 (87.65)	3,883 (87.26)	3,504 (88.08)	< 0.001
	≧45	1,041 (12.35)	567 (12.74)	474 (11.92)	
Bachelor degree	No	3,628 (43.05)	1952 (43.87)	1,676 (42.13)	0.109
	Yes	4,800 (56.95)	2,498 (56.13)	2,302 (57.87)	
Household income level	Low	3,587 (42.56)	1931 (43.39)	1,656 (41.63)	0.024
	Medium	2,149 (25.50)	1,156 (25.98)	993 (24.96)	
	High	2,692 (31.94)	1,363 (30.63)	1,329 (33.41)	
Self- rated health status	Fair or poor	233 (2.76)	91 (2.04)	142 (3.57)	< 0.001
	Good	1,336 (15.85)	617 (13.87)	719 (18.07)	
	Very good	6,859 (81.39)	3,742 (84.09)	3,117 (78.36)	
Chronic disease	No	6,745 (80.03)	3,759 (84.47)	2,986 (75.06)	< 0.001
	Yes	1,683 (19.97)	691 (15.53)	992 (24.94)	
COVID-19 infection (participants or family member)	No	7,643 (90.69)	4,299 (96.61)	3,344 (84.06)	< 0.001
	Yes	785 (9.31)	151 (3.39)	634 (15.94)	
Lost job due to covid-19	No	5,478 (65.00)	3,429 (77.06)	2049 (51.51)	< 0.001
	Yes	2,950 (35.00)	1,021 (22.94)	1929 (48.49)	
Food shortage	No	6,046 (71.74)	3,862 (86.79)	2,184 (54.90)	< 0.001
	Yes	2,382 (28.26)	588 (13.21)	1794 (45.10)	
Drug shortage	No	5,803 (68.85)	3,907 (87.80)	1896 (47.66)	< 0.001
	Yes	2,625 (31.15)	543 (12.20)	2082 (52.36)	
Degree of difficulty in daily household activities	No difficulty at all	2,602 (30.87)	1791 (40.25)	811 (20.39)	< 0.001
	Mild difficulties	3,803 (45.12)	2018 (45.35)	1785 (44.87)	
	Extreme difficulties	2023 (24.00)	641 (14.40)	1,382 (34.74)	
Perceived risks of infection	Low	1,560 (18.51)	964 (21.66)	596 (14.98)	< 0.001
	Medium	1738 (20.62)	914 (20.54)	824 (20.71)	
	High	5,130 (60.87)	2,572 (57.80)	2,558 (64.30)	

Chi-square test was used for balance checking.

### Self-care behavior outcomes

**Figure 1:**
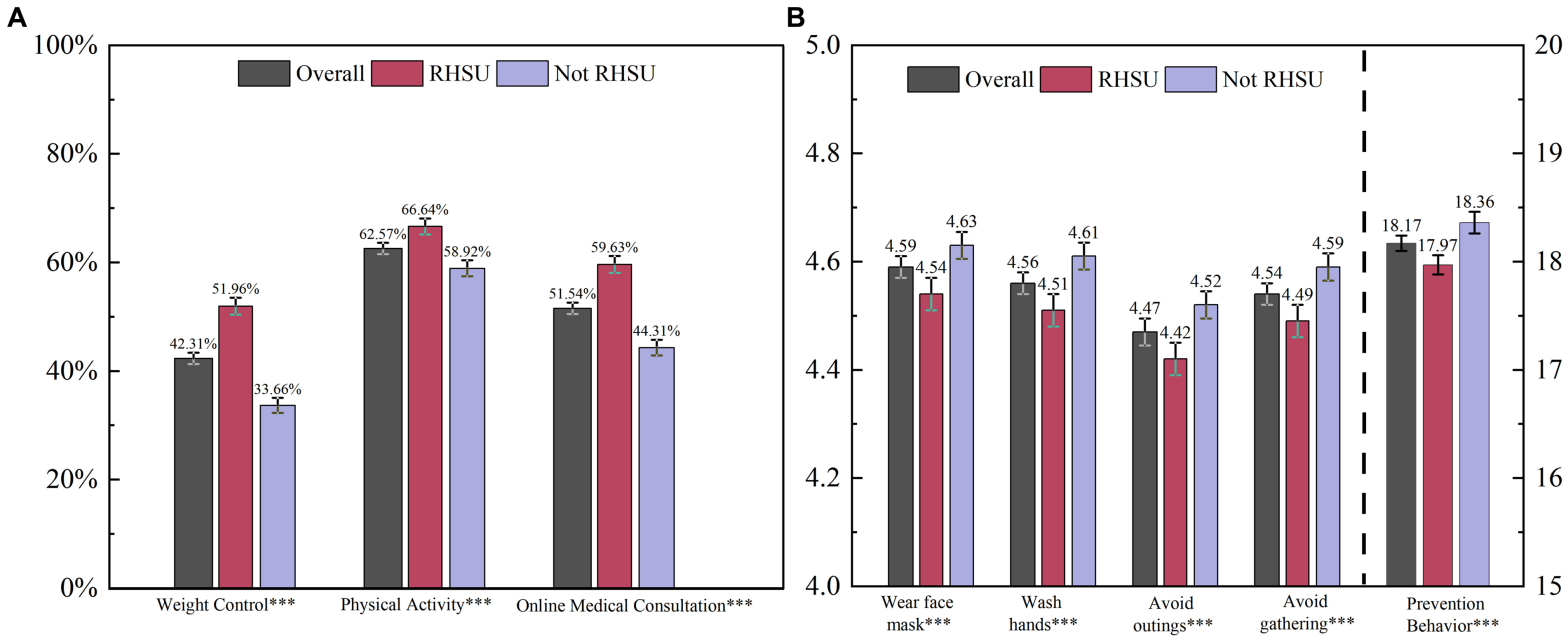
Self-care behavior outcomes between groups with or without restricted health service utilization among Chinese adults. **(A)** Shows the proportion of participants who engaged in weight control or physical activity and the proportion of above-average online medical consultation scores across the different groups. **(B)** Shows the mean of the total prevention behavior score and the score of each item. The total prevention behavior score ranges from 4 to 20, and the score of each item (i.e., wear face mask, wash hands, avoid outings, avoid gathering) ranges from 1 to 5. Higher scores mean better prevention behavior. The “Overall” represents the total sample, “Not RHSU” as the control group represents the participant’s routine medical care or medical consultations have not been restricted due to COVID-19 and “RHSU” represents the participant’s routine medical care or medical consultations have been restricted due to COVID-19. The error bars indicate a 95% confidence interval of the estimates. *** *p* < 0.01, ** *p* < 0.05, * *p* < 0.1.

The average total prevention behavior score for the groups in RHSU and control groups (Not RHSU) was 17.97 (SD = 3.54; 95% CI: 17.86–18.07; *p* < 0.001), 18.36 (SD = 3.34; 95% CI:18.26–18.45; *p* < 0.001), respectively (Figure 1). The mean Online medical consultation score was 1.51 (SD 1.34), used online consultation (52.63%), used video consultation (32.71), used telephone consultation service (44.23), used mail-order or personal delivery pharmacy (21.39%) and none used (36.22%). The data from Figure 1 showed that during the COVID-19 pandemic, 42.31% of participants engaged in weight control and 62.57% in physical activity. Compared to the control group, the RHSU group performed significantly better in weight control (proportion of responded “yes”: 51.96% vs. 33.66%; *p* < 0.001), physical activity (proportion of responded “yes”: 66.64% vs. 58.92%; *p* < 0.001) and online medical consultation (proportion of above-average score: 59.63% vs. 44.31%; *p* < 0.001).

The results of Multiple logistic regression analyses are shown in Table 2, we observed the RHSU was positively associated with prevention behavior (Coef = 0.16, 95% CI 0.003–0.321, *p* = 0.045), weight control (OR = 1.34, 95% CI 1.21–1.49, *p* < 0.001), physical activity (OR = 1.14,95% CI 1.03–1.27, *p* < 0.001), and online medical consultation (OR = 1.30, 95% CI 1.17–1.45, *p* < 0.001). From Table 2, we observed that participants aged 45 and over, female, married, non-chronic population, low-income groups, individuals who have not experienced COVID-19 infection (themself or family members), those who lived in low infection-risk areas, those whose daily household activities were not experienced difficulty due to COVID-19-related financial strain, and those with a perceived higher risk of infection were significantly more likely to report higher prevention behavior scores.

**Table 2:** Associations of RHSU with each self-care behavior outcomes among Chinese adults.

Variables	Prevention behavior^a^		Online medical consultation^b^		Weight control^b^		Physical activity^b^	
	Coef (95%CI)	*p*-value	OR (95%CI)	*p*-value	OR (95%CI)	*p*-value	OR (95%CI)	*p*-value
Restricted Health Service Utilization (Ref. = No)	-		-		-		-	
Yes	**0.16 (0.00,0.32)**	**0.045**	**1.30 (1.17,1.45)**	**< 0.001**	**1.34 (1.21,1.49)**	**< 0.001**	**1.14 (1.03,1.27)**	**0.014**
Region (Ref. = City)	-		-		-		-	
Rural	−0.09 (−0.26,0.08)	0.284	1.12 (0.00,1.26)	0.052	**0.86 (0.77,0.97)**	**0.011**	0.98 (0.88,1.10)	0.760
Town	−0.17 (−0.38,0.04)	0.106	1.04 (0.91,1.20)	0.561	**0.78 (0.68,0.89)**	**< 0.001**	0.95 (0.83,1.09)	0.461
Actual infection risk level of living area (Ref. = Low)	-		-		-		-	
Moderate	**−0.25 (−0.41,-0.09)**	**0.002**	**1.16 (1.04,1.29)**	**0.007**	0.95 (0.86,1.06)	0.384	0.90 (0.81,1.00)	0.053
High	**−0.23 (−0.45,-0.02)**	**0.036**	**1.89 (1.62,2.21)**	**< 0.001**	**1.24 (1.07,1.43)**	**0.004**	1.16 (0.99,1.35)	0.061
Married status (Ref. = Unmarried/divorced/widowed)	-		-		-		-	
Married	**0.58 (0.43,0.73)**	**< 0.001**	**1.46 (1.31,1.62)**	**< 0.001**	0.94 (0.85,1.05)	0.265	**1.35 (1.22,1.49)**	**< 0.001**
Sex (Ref. = Female)	-		-		-		-	
Male	**−0.38 (−0.52,-0.24)**	**< 0.001**	**1.38 (1.25,1.52)**	**< 0.001**	**0.86 (0.78,0.94)**	**< 0.001**	**1.32 (1.20,1.45)**	**< 0.001**
Age (years) (Ref. = 18–44)	-		-		-		-	
≧45	**0.32 (0.09,0.54)**	**0.005**	**0.35 (0.30,0.41)**	**< 0.001**	0.96 (0.83,1.12)	0.633	**0.84 (0.73,0.97)**	**0.020**
Bachelor degree (Ref. = No)	-		-		-		-	
Yes	0.12 (−0.03,0.26)	0.128	1.09 (0.99,1.21)	0.092	0.98 (0.88,1.08)	0.672	1.05 (0.95,1.17)	0.322
Household income Level (Ref. = Low)	-		-		-		-	
Medium	−0.16 (−0.34,0.02)	0.076	**1.40 (1.24,1.58)**	**< 0.001**	0.98 (0.87,1.10)	0.714	**1.39 (1.23,1.56)**	**< 0.001**
High	**−0.31 (−0.47,-0.14)**	**< 0.001**	**1.52 (1.36,1.71)**	**< 0.001**	**1.23 (1.10,1.37)**	**< 0.001**	**1.25 (1.12,1.40)**	**< 0.001**
Self- rated health status (Ref. = Fair or poor)	-		-		-		-	
Good	0.13 (−0.32,0.58)	0.576	1.02 (0.75,1.39)	0.915	1.31 (0.96,1.79)	0.086	**1.69 (1.25,2.29)**	**< 0.001**
Very good	0.30 (−0.12,0.73)	0.163	**1.86 (1.39,2.49)**	**< 0.001**	**1.83 (1.36,2.45)**	**< 0.001**	**4.17 (3.12,5.57)**	**< 0.001**
Chronic disease groups (Ref. = No)	-		-		-		-	
Yes	**−1.01 (−1.20,-0.83)**	**< 0.001**	**1.54 (1.35,1.76)**	**< 0.001**	**1.17 (1.04,1.33)**	**0.009**	0.93 (0.82,1.05)	0.247
COVID-19 infection (participants or family member)(Ref. = No)	-		-		-		-	
Yes	**−2.01 (−2.28,-1.74)**	**< 0.001**	1.03 (0.84,1.26)	0.786	**2.28 (1.88,2.76)**	**< 0.001**	**1.70 (1.38,2.09)**	**< 0.001**
Lost job due to COVID-19 (Ref. = No)	-		-		-		-	
Yes	0.10 (−0.07,0.27)	0.243	1.05 (0.93,1.18)	0.435	**1.34 (1.20,1.50)**	**< 0.001**	1.12 (0.99,1.26)	0.056
Food shortage (Ref. = No)	-		-		-		-	
Yes	−0.17 (−0.36,0.01)	0.070	**1.19 (1.04,1.35)**	**0.008**	**1.46 (1.30,1.65)**	**< 0.001**	**1.26 (1.11,1.43)**	**< 0.001**
Drug shortage (Ref. = No)	-		-		-		-	
Yes	0.00 (−0.18,0.18)	0.995	**1.56 (1.37,1.76)**	**< 0.001**	**1.43 (1.27,1.61)**	**< 0.001**	**1.37 (1.21,1.55)**	**< 0.001**
Degree of difficulty in daily household activities (Ref = No difficulty at all)	-		-		-		-	
Mild difficulties	**−0.31 (−0.48,-0.13)**	**< 0.001**	**1.94 (1.73,2.18)**	**< 0.001**	1.06 (0.94,1.19)	0.338	0.93 (0.83,1.04)	0.180
Extreme difficulties	**−0.76 (−1.00,-0.53)**	**< 0.001**	**2.38 (2.03,2.80)**	**< 0.001**	**1.31 (1.12,1.53)**	**< 0.001**	1.00 (0.85,1.18)	0.971
Perceived risks of infection (Ref. = Low)	-		-		-		-	
Medium	**0.35 (0.13,0.57)**	**< 0.001**	**0.60 (0.52,0.71)**	**< 0.001**	0.954 (0.81,1.10)	0.454	1.00 (0.86,1.16)	0.994
High	**1.25 (1.06,1.44)**	**< 0.001**	**0.38 (0.33,0.43)**	**< 0.001**	0.88 (0.78,1.00)	0.053	**0.85 (0.74,0.96)**	**0.011**

Coef., Coefficient; OR, Odds Ratio; CI, confidence interval. ^a^denotes continuous dependent variable, ^b^denotes binary dependent variable, statistically significant results are in bold (*p*<0.05).

The average overall score for online medical consultation and prevention behavior was 1.51 (SD = 1.34, 95% CI:1.48–1.54) and 18.17 (SD = 3.44, 95% CI:18.10–18.24). In Table 2, multi-variable regression analyses indicated that urban residents, females, high-income groups, chronic disease population, those who experienced job loss due to the pandemic (participants or family members), those who lived in high infection-risk areas, those with a history of COVID-19 infection (participants or family members), those with better self-rated health, those who experienced food or drug shortages, and those whose daily household activities experienced extreme difficulties due to COVID-19-related financial strains, were significantly more likely to engage in weight control. Meanwhile, the young group (18–44 years), male, married, medium and high-income groups, those with better self-rated health, those with a history of COVID-19 infection (participants or family members), and those who experienced food or drug shortage were significantly more likely to engage in physical exercise. Also, the results showed that the young group (18–44 years), male, married, medium and high-income groups, chronic disease population, those with very good self-rated health, those who lived in higher infection-risk areas, those experienced food or drug shortages, those whose daily household activities were experienced mild or extremely difficulties due to COVID-19-related financial strain, and those who perceived high risk of infection were significantly more likely to report above-average online medical consultation score.

### Subgroup analysis

**Figure 2:**
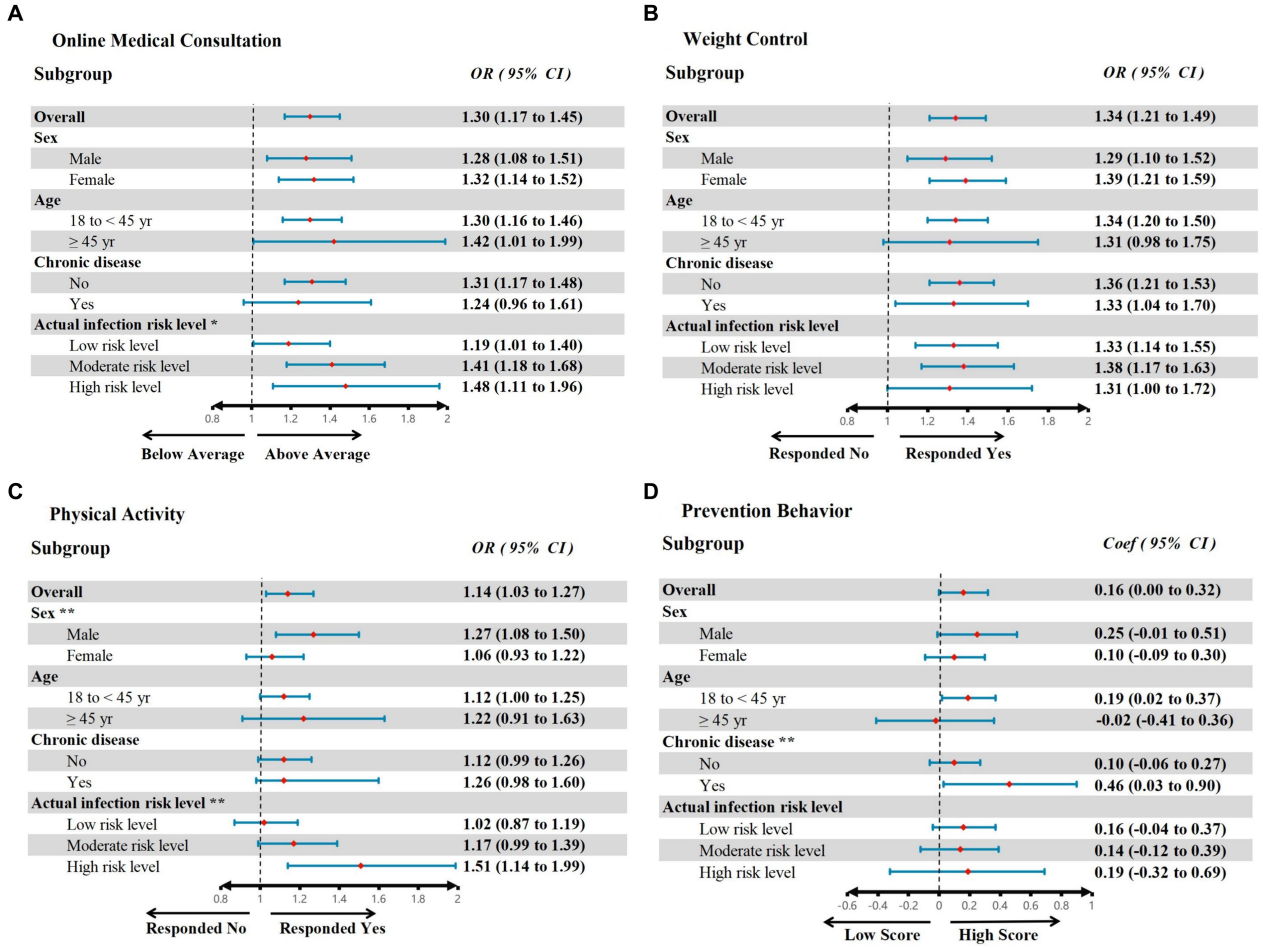
Associations of Restricted Health Service Utilization and Self-Care Behavior outcomes among subgroups. The red dots represent the observed mean value, and the blue lines represent the odds ratio **(A–C)** or coefficient **(D)** in the adjusted model; the CHOW test tested all outcomes of the subgroup analyses, ** Chow text *p* < 0.05, * Chow text *p* < 0.1. Adjust model control variables, i.e., age, sex, region, married status, actual infection risk level of living area, educational level, household income level, history of COVID-19 infection (participants or family member), chronic disease, self-rated health, food and drug shortage, lost job due to COVID-19, daily activities affected by COVID-19 related financial strain and perceived risk of infection. The Appendix File 2 shows details of multiple regression models for sex, age, chronic disease, and infection risk level of the living area subgroup.

Based on the relevant literature, we generated multiple logistic regression models that included all variables to conduct subgroup analyses among sex, age, chronic disease, and actual infection risk level of the respondent’s residence (41). According to the subgroup analysis shown in Figure 2, we observed significant positive associations between RHSU and four indicators of self-care behavior (weight control, physical activity, prevention behavior, and online medical consultation) in all populations. By conducting a Chow test on the coefficients of the subgroup regression, we found that male groups (Male, OR = 1.33, CI 1.06–1.1.67, *Chow text p* = 0.016) and participants living in high-risk areas were more likely to engage in physical activity (high infection-risk level, OR = 1.51, CI 1.03–1.27, *Chow text p* = 0.024) when RHSU occurred. Similarly, when both groups experienced RHSU, participants living in higher infection-risk areas were more likely to report above-average online medical consultation scores (high infection-risk level, OR = 1.48, CI 1.03–1.27, *Chow text p* = 0.078; moderate infection-risk level, OR = 1.41, CI 1.18–1.68, *Chow text p* = 0.098), In addition, the chronic disease group (Coef = 0.46, CI 0.03–0.90, *Chow text p* = 0.032) was more likely to report higher prevention behavior scores. Conversely, the relation between RHSU and weight control was not statistically different in subgroup analyses based on sex, age, chronic disease, and actual infection risk level of the respondent’s residence.

## Discussion

Since March 2020, the World Health Organization declared COVID-19 a global pandemic, and governments around the world imposed restrictions on the use of hospitals and outpatient services, eliminated all elective, routine and non-emergency patient procedures, implemented stricter physical distancing measures and transitioned to remote care to reallocate resources to the urgent care of patients with COVID-19 (42), which substantially disrupted individuals’ routine healthcare utilization. Globally, the Chinese government’s endeavor to contain the spread of the COVID-19 pandemic has been widely praised, which may have contributed to a more severe influence on individuals’ routine healthcare utilization during the early stages of the outbreak compared to other countries (9). To the best of our knowledge, this is the first study to explore the impact of health service utilization on self-care behavior among Chinese adult populations during the early COVID-19 pandemic based on a large sample covering 31 provinces in China. Furthermore, our study used two dimensions with four indicators to measure self-care behavior, explore the relationship between RUSH and self-care behavior, and examine vulnerable populations and subgroup differences through multilevel regression and subgroup analyses. Our findings from the COVID-19 pandemic may further elucidate the future policy development of self-care behavior in China, provide appropriate interventions to mitigate the health risks caused by RHSU and contribute to alleviating the burden on the public health system and its long-term benefits.

Firstly, the multivariate results supported much of what we anticipated earlier: the presence of RHSU was positively associated with all four kinds of self-care behavior in this study, and these associations remained significant even after adjusting for individual, environmental, and risk-perceptive control variables. Similar to the results of studies before (like medical services supply decline) and after the outbreak of the COVID-19 pandemic, these researchers also observed the association between social inequalities, pandemic-related changes, and individual health behavior (41–43). For instance, when individuals have potential health risks (including reduced accessibility to healthcare or deterioration of health status), this may stimulate the self-care activities and behaviors that were aimed at preventing or reducing health risks and optimizing health and quality of life (22, 44). Also, evidence from Anderson’s health behavior model likewise supports a relationship between the use of preventive health services and self-care ability in daily life (46). Regarding autonomous behavior, previous studies claim that there is an association between decreased healthcare utilization and increased leisure-time physical activity; this association remains after adjustment for socio-economic confounders (17), which is consistent with our findings. Research with adults during the COVID-19 pandemic suggests that self-isolation at home due to lockdown is associated with a lower level of physical activity and modifications in eating behavior (47, 48). Also, a classic literature review elucidated that individual age, health literacy, and self-rated health could indirectly influence the association between healthcare utilization and physical activity through mediation analyses (49). Based on the discussion above, we predict that the relationship between self-care behavior and RHSU will persist even after the COVID-19 pandemic subsides.

Secondly, even if there is a willingness to practice positive self-care behavior, some vulnerable people still perform worse in familiarizing self-care activities and building self-care behavior due to physical and psychological factors (26), which if ignored could make it difficult to implement the entire strategy of reducing the burden on public health through self-care behavior. For example, previous studies indicated that 30–49-year-old adults, those with a higher level of education, and those who were employed and had a high income were more knowledgeable and better able to take appropriate measures to prevent the spread of COVID-19 (35, 50). However, our study observed that participants in the survey had generally high scores on prevention behaviors related to COVID-19, and these differences between populations were not apparent compared to other studies, which may be attributed to the Chinese government’s extensive publicity and appropriate supervision of interrupting the spread of the disease in the early stages of the COVID-19 pandemic (51).

Similarly, our study indicated significant correlations in online medical consultation across age, gender, income, married status, health status, and infection risk perception, those findings align with previous studies (27, 52). It is not surprising that social distance, isolation, and hospital restrictions forced chronic patients to re-organize their routine care through temporary in-person visits during the pandemic, this led to a widespread and significant increase in telemedicine utilization (3). Also, patients with chronic disease may avoid in-person visits to hospitals, clinics, and emergency departments for fear of exposure to potential COVID-19-infected patients (3, 53), thereby preferring to implement telemedicine or seek online medical advice. However, our results showed that middle-aged and older adults showed relatively weak performance in medical consultation compared to younger adults. In fact, despite research examining age differences in self-care during the COVID-19 pandemic has been inconclusive, previous studies on the digital divide demonstrated that people aged above 65 years are less likely than the younger generation to have had the chance to familiarize themselves with ICT either at school or at work, combined with the cognitive, motor, digital gap and sensory decline associated with aging, and older adults face more barriers to and challenges in using online technology for health than their younger counterparts (54). The present studies claimed that this digital divide created by digital technologies might widen social inequalities by alienating disadvantaged groups that do not have access to digital resources (55, 56). Thus, we recommend that particular attention be paid to the older adult when future discussions on critical issues surrounding the promotion of online medical consultations include quality of care (57), communication and language barriers (58), and patient satisfaction (43)^.^

Thirdly, our study also extended the existing literature by the correlations between restricted health service utilization and self-care behavior, which were quantified and compared by subgroup analysis. Based on this, we found that external and intrinsic risk factors had a significant effect on some type of self-care behavior. For example, residents living in the higher infection-risk areas were more likely to report above-average online medical consultation scores or engage in physical activities when they experienced RHSU. The strength of this association varies significantly with the infection risk level of the living area. These findings are also consistent with the argument for behavioral change theory since high infection-risk living environments created additional barriers (e.g., extra healthcare costs, and psychological stress) to illness prevention and health maintenance. Therefore, except for inequities in health service utilization, the risk perception (41) and actual infection risk related to the pandemic (44) may be critical indicators of health behavior choices. A possible explanation from psychology is that the higher the risk an individual perceives, the more motivated they might be to engage in protective behaviors (53). Despite this, both external and intrinsic risk factors need to be considered for personal characteristics, previous research highlighted that not everyone responds to health risks similarly and that risk perception alone does not explain health behavior (59). For instance, the self-care behavior of chronic disease patients is associated with a high perceived susceptibility to disorders; they may tend to avoid in-person visits to hospitals, clinics, and emergency departments for fear of exposure to potential COVID-19 (43). Likewise, older adults are more vulnerable to COVID-19 infection, have a worse prognosis after infection, and have a higher risk of getting one or more non-communicable diseases, so that they may experience heightened levels of instilled fear of COVID-19 exposure during in-person medical services (3). In general, caution must be exercised in the following discussion of these findings.

In contrast, no significant associations were found in the model of weight control among subgroups analysis, which means that the strength of the association between RHSU and weight control was not affected by age, chronic diseases, and actual infection risk level of the respondent’s residence. Still, we found several reasons that may confound our findings by searching the literature. A study from *Obesity* has suggested that social closure measures may have a wide-ranging effect, making it more difficult for many people to adopt weight gain protective behaviors (60). It was widespread for people to experience barriers to diet and healthy eating during the COVID-19 pandemic lockdown (43, 47) (e.g., food shortage, lacking motivation and control around food). Also, some mental health issues, such as anxiety, stress, and poor mood, were found to be risk factors for obesity during the pandemic (61). Collectively, the factors mentioned above may make it more difficult to distinguish differences in weight control behaviors among populations.

At last, given that individuals who experienced restricted health service utilization were more actively engaged in self-care behavior, our study examines the interaction of health service inequalities and potential risk factors on self-care behavior during the pandemic. It highlights the necessity of promoting self-care behavior when in response to health service restrictions, especially for vulnerable individuals (62). Meanwhile, in view of the various benefits of self-care behavior, we need more comprehensive policies to encourage individuals to develop self-care behavior. Both primary healthcare institutions and community service organizations should strengthen the strategic support related to self-care promotion and implementation for the public regarding personal health literacy development, public healthcare services, online healthcare services, continuing medical education, etc.

From the perspective of health care system reform, addressing current barriers around self-care in terms of applicability, developmental disequilibrium, standardization, supervision, service coverage, and digital divide caused by technological advances will help more people maintain their health through self-care activities effectively; it not only effectively responds the issue of restricted health service utilization, but also contributes to alleviate the burden on the public health system and its long-term benefit (63). Indubitably, there is a pressing need to strengthen the development of self-care systems in China.

Our study includes several fundamental limitations that must be acknowledged and addressed in future studies. Given the cross-sectional nature of this study, one of the main limitations was that there was no baseline response rate before the pandemic and no available data on participants’ previous self-care behavior; neither can make definitive statements about causality in regression analyses. Second, this study assessed outcome variables by employing a few single-item scales; also, only three dimensions were collected to describe the performance of self-care behavior. Therefore, future research should determine variables more comprehensively by using multiple scales to provide more conclusive evidence on the predictive validity of self-care behavior. Third, participants were recruited using a snowball sampling method through social media; the advantage of this method is that a large number of samples can be collected quickly. However, many participants were well-educated and below 40 years old, leading to a particular bias in the results, which made it difficult to identify more subgroups with significant differences. Fourth, the subgroup analyses in this study did not use more demographic categories, such as region, economic status, and education level; these subgroup differences require deeper exploration in the future.

However, this study has some innovative findings. First, our findings extend the existing literature by exploring the impact of restricted health service utilization on self-care behavior in a large sample covering all provinces in mainland China. Based on the RAA theory, our study may find some motivations and factors for self-care behavior change; these may provide some references for promoting the effectiveness of self-care behavior to reduce the burden on the health service system. Second, in contrast to previous studies mainly focusing on the impact of inequalities in health service accessibility or quality on self-care behavior in the context of regional economic disparities, our study contributes to the existing knowledge base by investigating the relationship between restricted health service utilization and self-care behavior during a large scale infectious disease crisis. Third, we also take into account differences in risk perception when exploring the impact of inequalities in health service utilization on the outcome variables, so we tried to describe and compare the association between the RHSU and self-care behavior among the sex, age, chronic disease and high, middle or low risk of infections living area subgroups, it is effective in terms of filling the gap in the relevant literature.

## Conclusion

The COVID-19 pandemic dramatically impacted health service utilization across China (9), and this study sheds light on the restricted health service utilization may predict more positive self-care behavior during the pandemic and the differential presentation of this association between subgroups of sex, age, chronic disease, and actual infection risk level of the residence area. Based on our results and current research findings, we believe that the correlation between restricted health service utilization and self-care behavior will persist as the COVID-19 pandemic subsides. Thus, we need further research into the mechanisms of self-care behavior, as well as continuing to address self-care knowledge gaps and improve outcomes. Given the many existing challenges to our vision, it is necessary to drive the development of policies related to self-care behavior to raise self-care as a vital element in general health and healthcare, which not only effectively responds the adverse health outcomes from restricted health service utilization and future public health crisis, but also contributes to alleviating the burden on the healthcare system and its long-term benefits.

## Data availability statement

The data analyzed in this study is subject to the following licenses/restrictions: the datasets presented in this article are not readily available because ethics restrictions. Requests to access these datasets should be directed to youfawang@gmail.com.

## Ethics statement

The studies involving humans were approved by the Institutional Review Committees of the Xi’an Jiaotong University, China (approval number 2020-1172). The studies were conducted in accordance with the local legislation and institutional requirements. Written informed consent for participation in this study was provided by the participants’ legal guardians/next of kin.

## Author contributions

ZW: Conceptualization, Data curation, Formal analysis, Resources, Software, Writing – original draft, Writing – review & editing. ZZ: Data curation, Formal analysis, Funding acquisition, Writing – review & editing, Supervision. GL: Investigation, Methodology, Project administration, Writing – original draft. JL: Resources, Writing – review & editing, Formal analysis, Investigation. XZ: Methodology, Writing – review & editing, Resources, Validation. XF: Data curation, Investigation, Writing – review & editing, Methodology. SL: Conceptualization, Data curation, Investigation, Writing – review & editing. YW: Conceptualization, Data curation, Investigation, Writing – review & editing.
