# Supplementary material for: Restricted health service utilization and subsequent positive self-care behavior during the early COVID-19 pandemic in China
Source: Front Public Health. 2024 Jul 9;12:1398271. doi: 10.3389/fpubh.2024.1398271 (PMC11263186; doi:10.3389/fpubh.2024.1398271)
Supplement: Supplementary file 1 [file Table_1.doc]

**The multiple regression models of self-care behavior among sex, age, chronic disease , infection risk level of living area subgroups.**

Table s1 The multiple regression models among sex, age, chronic disease , infection risk level of living area subgroups. (Prevention Behavior)

| Variables | | **Prevention Behavior** | **Prevention Behavior** | **Prevention Behavior** | **Prevention Behavior** | **Prevention Behavior** | **Prevention Behavior** | **Prevention Behavior** | **Prevention Behavior** | **Prevention Behavior** |
| --- | --- | --- | --- | --- | --- | --- | --- | --- | --- | --- |
|  |  | Male  N=3694 | Female  N=4734 | NO-Chronic disease Groups N=6745 | Chronic disease Groups N=1683 | Age=18-44 N=7387 | Age≧45 N=1041 | Regional Risk level= Low N=3787 | Regional Risk level=Medium N=3065 | Regional Risk level=High N=1576 |
|  |  | *Coef(95%CI)* | *Coef(95%CI)* | *Coef(95%CI)* | *Coef(95%CI)* | *Coef(95%CI)* | *Coef(95%CI)* | *Coef(95%CI)* | *Coef(95%CI)* | *Coef(95%CI)* |
| Restricted health service utilization | No | - | - | - | - | - | - | - | - | - |
|  | Yes | 0.25(-0.01,0.51)* | 0.10(-0.09,0.30) | 0.10(-0.06,0.27) | 0.46(0.03,0.90)** | 0.19(0.02,0.37)** | -0.02(-0.41,0.36) | 0.16(-0.04,0.37) | 0.14(-0.12,0.39) | 0.19（-0.32,0.69） |
| Region | City | - | - | - | - | - | - | - | - | - |
|  | Rural | -0.16(-0.44,0.13) | -0.64(-0.28,0.15) | -0.07(-0.25,0.11) | -0.15(-0.61,0.31) | -0.04(-0.23,0.14) | -0.54(-0.98,-0.11)** | -0.21(-0.43,0.00)* | 0.13(-0.14,0.41) | -0.41（-0.95,0.13） |
|  | Town | -0.28(-0.62,0.06) | -0.08(-0.33,1.18) | -0.18(-0.39,0.04) | -0.10(-0.67,0.47) | -0.15(-0.37,0.07) | -0.42(-1.03,0.18) | -0.13（-0.51,0.14） | 0.02(-0.31,0.35) | -0.57（-1.14,0.00）** |
| Risk level of  living area | Low | - | - | - | - | - | - | low vs med + high Chow text p=0.47 | low vs med Chow text p=0.40 | low vs high Chow text p=0.416 |
|  | Medium | -0.08(-0.34,0.18) | -0.36(-0.42,-0.17)*** | -0.23(-0.39，-0.06)*** | -0.32(-0.76,0.12) | -0.23(-0.40,-0.06)*** | -0.48(0.87,-0.08)** |  |  |  |
|  | High | -0.12(-0.45,0.22) | -0.31(-060,-0.02)** | -0.27(-0.51,-0.04)** | 0.06(-0.60,0.48) | -0.21(-0.44,0.02)* | -0.33(-0.94,0.29) |  |  |  |
| Married status | Unmarried / divorced / widowed | - | - | - | - | - | - | - | - | - |
|  | Married | 0.68(0.45,0.92)*** | 0.49(0.28,0.69)*** | 0.52(0.36,0.68)*** | 0.54(0.12,0.96)** | 0.60(0.44,0.76)*** | 0.42(-0.23,1.08) | 0.46（0.26,0.67）*** | 0.59(0.34,0.84)*** | 0.65（0.22,1.08）*** |
| Sex | Female |  | Chow text p=0.144 | - | - | - | - | - | - | - |
|  | Male |  |  | -0.36(-0.51,0.21)*** | -0.61(-0.99,-0.23)*** | -0.39(-0.54,-0.23)*** | -0.26(0.62,0.10) | -0.40（-0.59,-0.22）*** | -0.28(0.51,-0.05)** | -0.47（-0.89,-0.04）** |
| Age (years) | 18-44 | - | - | - | - |  |  | - | - | - |
|  | ≧45 | 0.40(0.03,0.77)** | 0.25(-0.02,0.52)* | 0.06(-0.19,0.30) | 0.68(0.18,1.17)*** |  | Chow text p=0.186 | 0.22(-0.04,0.48)* | 0.26(-0.13,0.66) | 0.83（0.05,1.60）** |
| bachelor degree | No | - | - | - | - | - | - | - | - | - |
|  | Yes | 0.09(-0.14,0.33) | 0.11(-0.08,0.31) | -0.01(-0.17,0.14) | 0.53(0.14,0.92)*** | 0.14(-0.03,0.30)* | -0.15(-0.56,0.25) | -0.22（-0.42，-0.02）** | 0.27(0.03,0.52)** | 0.56（0.14,0.98）*** |
| Household income level | Low | - | - | - | - | - | - | - | - | - |
|  | Medium | -0.10(-0.38,0.18) | -0.20(-0.43,0.03)* | -0.10(-0.28,0.08) | -0.39(-0.89,0.10) | -0.16(-0.35,0.03)* | -0.06(-0.52,0.40) | -0.21（-0.43,0.02）* | -0.06(-0.35,0.24) | -0.26（-0.82,0.31） |
|  | High | -0.29(-0.59,-0.02)** | -0.32(-0.52,-0.11)*** | -0.15(-0.32,0.02)* | -0.88(-1.31,-0.45)*** | -0.31(-0.49,-0.13)*** | -0.20(-0.62,0.22) | -0.20（-0.42,0.01）* | -0.38(-0.65,-0.11)*** | -0.38（-0.85,0.09） |
| Self- rated health status | Fair or poor | - | - | - | - | - | - | - | - | - |
|  | good | 0.41(-0.38,1.19) | 0.01(-0.53,0.54) | -0.12(-0.68,0.44) | 0.73(-0.09,1.55)* | 0.13(-0.37,0.62) | -0.10(-1.08,0.89) | 0.38（-0.23,0.99) | -0.12(-0.83,0.59) | -0.01（-1.28,1.27） |
|  | very good | 0.80(0.06,1.54)** | 0.00(-0.51,0.51) | 0.16(-0.37,0.69) | 0.84(0.06,1.61)** | 0.40(-0.07,0.87)* | -0.31(-1.28,0.66) | 0.45(-0.13,1.03) | 0.00(-0.68,0.67) | 0.55（-0.59,1.70） |
| Chronic disease | No | - | - |  |  | - | - | - | - | - |
|  | Yes | -1.08(-1.36,-0.80)** | -0.94(-1.18,-0.70)*** |  | Chow text p=0.032 | -1.14(-1.34,-0.93)*** | -0.42(-0.80,-0.04)** | -0.77(-1.02,0.52)*** | -1.02(-1.32,0.72)*** | -1.17（-1.63，-0.70）*** |
| COVID-19 infection (participants or family member) | No | - | - | - | - | - | - | - | - | - |
|  | Yes | -2.07(-2.45,-1.69)*** | -1.92(-2.31,-1.53)*** | -1.47(-1.79,-1.15)*** | -2.69(-3.25,-2.14)*** | -2.07(-2.35,-1.78)*** | -0.32(-1.32,0.69) | -3.38(-3.85,-2.91)*** | -1.83(-2.34,-1.31)*** | -1.41(-1.93,-0.88)*** |
| Lost job due to COVID-19 | No | - | - | - | - | - | - | - | - | - |
|  | Yes | 0.15(-0.12,0.42) | 0.04(-1.18,-0.70) | 0.00(-0.18,0.18) | 0.46(0.02,0.90)** | 0.06(-0.12,0.24) | 0.40(-0.08,0.88) | -0.02(-0.25,0.22) | -0.06(-0.32,0.21) | 0.47(-0.01,0.95)** |
| Food shortage | No | - | - | - | - | - | - | - | - | - |
|  | Yes | -0.25(-0.54,0.04)* | -0.11(-0.34,0.13) | -0.14(-0.33,0.06) | -0.31(-0.78,0.16) | -0.21(-0.41,-0.01)** | 0.22(-0.31,0.74) | -0.13(-0.40,0.13) | -0.20(-0.49,0.09） | -0.09(-0.85,0.39) |
| Drug shortage | No | - | - | - | - | - | - | - | - | - |
|  | Yes | 0.09(-0.20,0.38) | -0.08(-0.31,0.16) | -0.14(-0.33,0.05) | 0.40(-0.07,0.87)* | 0.01(-0.18,0.21) | -0.23(-0.82,0.26) | -0.43(-0.68,-0.18)*** | 0.25（-0.04,0.53）* | 0.32(-0.18,0.83) |
| Daily activities affected by COVID-19-related financial strain | No difficulty | - | - | - | - | - | - | - | - | - |
|  | Mild difficulties | -0.44(-0.73,-0.15)*** | -0.21(-0.42,-0.01)** | -0.28(-0.46,-0.10)*** | -0.38(-0.89,0.12) | -0.37(-0.55,-0.18)*** | -0.05(-0.46,0.35) | -0.21(-0.42,-0.01)** | -0.32(-0.61，-0.03）** | 0.00(-0.76,0.77) |
|  | Extreme difficulties | -0.85(-1.22,-0.48)*** | -0.69(-0.99,-0.38)*** | -0.75(-1.00,-0.50)*** | -0.93(-1.54,-0.32)*** | -0.79(-1.04,-0.54)*** | -0.79(-1.47,-0.11)** | -0.59(-0.93,-0.26)*** | -0.67(-1.06,-0.28)*** | -0.67(-1.42,0.09) |
| Perceived risks of infection | Low | - | - | - | - | - | - | - |  | - |
|  | Medium | 0.22(-0.14,0.58) | 0.43(0.15,0.72)*** | 0.22(-0.02,0.46)* | 0.74(0.18,1.31)** | 0.33(0.97,0.57)*** | 0.63(-0.01,1.26)* | 0.30(0.02,0.58)** | 0.63(0.26,1.01)*** | 0.01(-0.64,0.65) |
|  | High | 1.42(1.12,1.72)*** | 1.11(0.86,1.35)*** | 0.97(0.77,1.17)*** | 2.30(1.81,2.80)*** | 1.29(1.08,1.49)*** | 1.06(0.53,1.59)*** | 0.68(0.44,0.93)*** | 1.75（1.41,2.10）*** | 1.88(1.35,2.41)*** |

Note: Coef: Coefficient, CI : confidence interval, ***p<0.01, **p<0.05,*p<0.1,

Table s2 The multiple regression models among sex, age, chronic disease , infection risk level of living area subgroups. (Online Medical consultation)

| Variables | | **Online Medical Consultation** | **Online Medical Consultation** | **Online Medical Consultation** | **Online Medical Consultation** | **Online Medical Consultation** | **Online Medical Consultation** | **Online Medical Consultation** | **Online Medical Consultation** | **Online Medical Consultation** |
| --- | --- | --- | --- | --- | --- | --- | --- | --- | --- | --- |
|  |  | Male  N=3694 | Female  N=4734 | NO-Chronic disease Groups N=6745 | Chronic disease Groups N=1683 | Age=18-44 N=7387 | Age≧45 N=1041 | Risk level= Low N=3787 | level=Midden  N=3065 | Risk level=High N=1576 |
|  |  | OR(95%CI) | OR(95%CI) | OR(95%CI) | OR(95%CI) | OR(95%CI) | OR(95%CI) | OR(95%CI) | OR(95%CI) | OR(95%CI) |
| Restricted health service utilization | No | - | - | - | - | - | - | - | - | - |
|  | Yes | 1.28(1.08,1.51)  *** | 1.32（1.14,1.52）*** | 1.31(1.17,1.48)*** | 1.24（0.96,1.61）* | 1.30(1.16，1.46)*** | 1.42（1.01,1.99）** | 1.19(1.01,1.40)** | 1.41(1.18,1.68)*** | 1.48（1.11,1.96）*** |
| Region | City | - | - | - | - | - | - | - | - | - |
|  | Rural | 1.04(0.87,1.26) | 1.10（0.91,1.32） | 1.05(0.92,1.19) | 1.58（1.19,2.09）*** | 1.10（0.97,1.25） | 1.36（0.94,1.98） | 1.07(0.90,1.27) | 1.16(0.96,1.41) | 1.16（0.84,1.59） |
|  | Town | 0.98(0.79,1.22) | 1.18（1.01,1.38）** | 0.99(0.85,1.16)* | 1.40（0.98,1.98）* | 1.03（0.89,1.19） | 1.25（0.76,2.05） | 1.01(0.81,1.26) | 1.06(0.85,1.34) | 1.09（0.78,1.53） |
| Risk level of  living area | Low | - | - | - | - | - | - | low vs mid + high Chow text p=0.038 | low vs mid Chow text p=0.098 | low vs high Chow text p=0.078 |
|  | Medium | 1.30(1.10,1.53)*** | 1.06（0.92,1.22） | 1.12(0.00,1.26)** | 1.36（1.04,1.76）** | 1.16（1.04,1.30）*** | 1.07（0.76,1.51） |  |  |  |
|  | High | 2.32(1.85,2.92)*** | 1.59（1.29,1.96）*** | 1.94(1.63,2.31)*** | 1.75（1.25,2.46）*** | 1.83（1.56,2.15）*** | 2.41（1.48,3.93）*** |  |  |  |
| Married status | Unmarried / divorced / widowed | - | - | - | - | - | - | - | - | - |
|  | Married | 1.23(1.05,1.43)*** | 1.77（1.52,2.05）*** | 1.54(1.37,1.73)*** | 1.06（0.82,1.38） | 1.47（1.32,1.64）*** | 1.32（0.73,2.39） | 1.54(1.30,1.81)*** | 1.52(1.28,1.81)*** | 1.27（0.98,1.64）* |
| Sex | Female |  | Chow text p=0.404 | - | - | - | - | - | - | - |
|  | Male |  |  | 1.43(1.28,1.59)*** | 1.15(0.91,1.45) | 1.45 (1.31,1.61)*** | 0.90（0.66,1.24） | 1.17 (1.01,1.35)** | 1.51（1.29,1.78)*** | 1.81（1.41,2.33）*** |
| Age (years) | 18-44 | - | - | - | - | - | - | - | - | - |
|  | ≧45 | 0.28(0.22,0.37)*** | 0.42（0.34，0.52）*** | 0.40(0.33,0.48)*** | 0.27（0.20,0.37）*** |  | Chow text p=0.274 | 0.34(0.27,0.43) | 0.30（0.22,0.40）*** | 0.52（0.34,0.80）*** |
| bachelor degree | No | - | - | - | - | - | ^ | - | - | - |
|  | Yes | 1.07(0.91,1.25) | 1.77（1.52,2.05）*** | 1.09(0.97,1.22) | 1.16（0.91,1.48） | 1.11（0.99,1.24）* | 1.18（0.83,1.69） | 1.06(0.91,1.24) | 1.07（0.90,1.27） | 1.32（1.03,1.70）** |
| Household income level | Low | - | - | - | - | - | - | - | - | - |
|  | Medium | 1.47(1.122,1.76)*** | 1.33（1.13,1.57）*** | 1.36(1.19,1.55)*** | 1.59（1.17,2.15）*** | 1.45（1.28,1.65）*** | 0.93（0.61,1.43） | 1.22(1.03,1.46)** | 1.71（1.40,2.10）*** | 1.24（0.90,1.72） |
|  | High | 1.48(1.24,1.77)*** | 1.51（1.30,1.75）*** | 1.50(1.33,1.71)*** | 1.59（1.22,2.07）*** | 1.47（1.30,1.65）*** | 1.93（1.37,2.72）*** | 1.55(1.31,1.84)*** | 1.41（1.17,1.70）*** | 1.68（1.28,2.22）*** |
| Self- rated health status | Fair or poor | - | - | - | - | - | - | - | - | - |
|  | good | 0.99(0.60,1.64) | 1.04(0.70,1.55) | 1.31(0.86,2.02) | 0.80（0.50,1.31） | 1.02（0.83,1.43） | 0.99（0.44,2.27） | 0.79(0.48,1.29) | 1.60（0.95,2.68）* | 0.88（0.43,1.80）* |
|  | very good | 1.74(1.08,2.78)** | 1.91(1.31,2.78)*** | 2.49(1.66,3.74)*** | 1.29（0.81,2.03） | 1.96（1.43,2.69）*** | 1.18（0.53,2.64） | 1.53(0.96,2.44)* | 2.95（1.80,4.84）*** | 1.32（0.70,2.52） |
| Chronic disease | No | - | - | - | ^ | - | - | - | - | - |
|  | Yes | 1.35,1.12,1.64)*** | 1.68(1.41,2.01)*** | **-** | Chow text p=0.35 | 1.69（1.46,1.95）*** | 1.03（0.74,1.44） | 1.45(1.19,1.78)*** | 1.89（1.52,2.35）*** | 1.32（0.99,1.76）* |
| COVID-19 infection (participants or family member) | No | - | - | - | - | - | - | - | - | - |
|  | Yes | 1.16(0.87,1.54) | 0.88(0.65,1.19) | 1.19(0.93,1.54) | 0.74（0.51,1.06）* | 1.02（0.83,1.26） | 0.96（0.41,2.22） | 1.31(0.88,1.94) | 1.01（0.69,1.49） | 1.02（0.73,1.42） |
| Lost job due to COVID-19 | No | - | - | - | - | - | - | - | - | - |
|  | Yes | 1.04(0.87,1.54) | 1.04(0.89,1.22) | 1.03(0.90,1.17) | 1.15（0.88,1.50） | 1.04（0.92,1.18） | 1.03（0.70,1.52） | 1.21(1.01,1.46)** | 0.85（0.71,1.02）* | 1.19（0.90,1.56） |
| Food shortage | No | - | - | - | - | - | - | - | - | - |
|  | Yes | 1.25(1.03,1.52)** | 1.14(0.96,1.35) | 1.15(0.99,1.32)* | 1.38（1.04,1.84）** | 1.20（1.05,1.37）*** | 1.04（0.38,1.59） | 1.19(0.96,1.46) | 1.32（1.08,1.62）*** | 0.94（0.71,1.25） |
| Drug shortage | No | - | - | - | - | - | - | - | - | - |
|  | Yes | 1.79(1.48,2.16)*** | 1.42(1.20,1.68)*** | 1.54(1.34,1.78)*** | 1.62（1.22,2.15）*** | 1.56（1.37,1.78）*** | 1.52（1.02,2.28）** | 1.55(1.28,1.89)*** | 1.56（1.28,1.90）*** | 1.62（1.20,2.17）*** |
| Daily activities affected by COVID-19-related financial strain | No difficulty | - | - | - | - | - | - | - | - | - |
|  | Mild difficulties | 1.81(1.51,2.18)*** | 2.08(1.78,2.43)*** | 2.09（1.83,2.37）*** | 1.33（0.99,1.78）* | 1.95(1.72,2.21)*** | 1.69（1.18,2.42)*** | 1.80(1.54,2.11)*** | 2.24（1.83,2.75）*** | 1.78（1.18,2.68）*** |
|  | Extreme difficulties | 1.95(1.53,2.48)*** | 2.88(2.30,3.59)*** | 2.50（2.08,2.99）*** | 1.80(1.25,2.60)** | 2.34（1.98,2.78）*** | 2.77(1.60,4.78)*** | 2.55(1.96,3.30)*** | 2.67（2.03,3.50）*** | 2.07（1.37,3.12）*** |
| Perceived risks of infection | Low | - | - | - | - | - | - | - |  | - |
|  | Medium | 0.71(0.55,0.90)*** | 0.54(0.44,0.66)*** | 0.59（0.50,0.70）*** | 0.70（0.48,1.00）** | 0.63（0.53,0.74）*** | 0.42(0.25,0.70)*** | 0.53(0.42,0.66)*** | 0.56（0.43,0.73）*** | 1.19（0.79,1.79） |
|  | High | 0.51(0.42,0.63)*** | 0.30(0.25,0.36)*** | 0.37（0.32,0.44）*** | 0.39（0.28,0.53）*** | 0.39（0.34,0.45）*** | 0.28(0.18,0.42)*** | 0.35(0.29,0.43)*** | 0.31（0.24,0.39）*** | 0.80（0.58,1.10） |

Note: OR : Odds Ratio , CI : confidence interval, ***p<0.01, **p<0.05,*p<0.1,

Table s3 The multiple regression models among age, chronic disease , infection risk level of living area subgroups. (Weight Control)

| Variables | | **Weight Control** | **Weight Control** | **Weight Control** | **Weight Control** | **Weight Control** | **Weight Control** | **Weight Control** | **Weight Control** | **Weight Control** |
| --- | --- | --- | --- | --- | --- | --- | --- | --- | --- | --- |
|  |  | Male  N=3694 | Female  N=4734 | Responded “yes” No-Chronic disease Groups N=6745 | Responded “yes” Chronic disease Groups N=1683 | Responded “yes” Age=18-44 N=7387 | Responded “yes” age≧45 N=1041 | Responded “yes” Regional Risk level= Low N=3787 | Responded “yes” Regional Risk level=Medium N=3065 | Responded “yes” Regional Risk level=High N=1576 |
|  |  | OR(95%CI) | OR(95%CI) | OR(95%CI) | OR(95%CI) | OR(95%CI) | OR(95%CI) | OR(95%CI) | OR(95%CI) | OR(95%CI) |
| Restricted health service utilization | No | - | - | - | - | - | - | - | - | - |
|  | Yes | 1.29(1.10,1.52)*** | 1.39(1.21,1.59)*** | 1.36(1.21,1.53)*** | 1.33（1.04,1.70）*** | 1.34(1.20,1.50)*** | 1.31（0.98,1.75）* | 1.33（1.14,1.55）*** | 1.38（1.17,1.63）*** | 1.31(1.00,1.72)** |
| Region | City | - | - | - | - | - | - | - | - | - |
|  | Rural | 0.90(0.75,1.08) | 0.84(0.73,0.96)** | 0.88(0.78,1.00)** | 0.78（0.60,1.02）* | 0.85(0.75,0.96)*** | 0.97（0.70,1.35） | 0.94（0.80,1.11） | 0.82（0.68,0.98）** | 0.75(0.55,1.01)* |
|  | Town | 0.88(0.70,1.09) | 0.73(0.61,0.88)*** | 0.74(0.64,87)*** | 0.94（0.67,1.31） | 0.80(0.69,0.92)*** | 0.57(0.35,0.93)** | 0.85（0.68,1.05） | 0.82（0.57,0.90）*** | 0.79(0.57,1.09) |
| Risk level of  living area | Low | - | - | - | - | - | - | low vs med + high Chow text p=0.37 | low vs med Chow text p=0.37 | low vs high Chow text p=0.45 |
|  | Medium | 1.16(0.98,1.67)* | 0.85(0.74,0.97)** | 0.95(0.83,1.08) | 0.94（0.73,1.20） | 0.93(0.84,1.05) | 1.08（0.80,1.46） |  |  |  |
|  | High | 1.38(1.11,1.71)*** | 1.16(0.95,1.51) | 1.19(1.06,1.35）*** | 1.25（0.91,1.72） | 1.22(1.05,1.42)** | 1.25（0.79,2.00） |  |  |  |
| Married status | Unmarried / divorced / widowed | - | - | - | - | - | - | - | - | - |
|  | Married | 1.03(0.88,1.20) | 0.91(0.78,1.05) | 0.92（0.82,1.03） | 1.06（0.83,1.36） | 0.94(0.84,1.04) | 0.99（0.60,1.64） | 0.98（0.84,1.15） | 0.83（0.71,0.98）** | 1.21(0.94,1.55) |
| Sex | Female | - | Chow text p=0.294 | - | - | - | - | - | - | - |
|  | Male |  |  | 0.81(0.73,0.90)*** | 1.09(0.88,1.37) | 0.84 (0.76,0.93)*** | 0.94 (0.72,1.24) | 0.71（0.62,0.82）*** | 1.00（0.85,1.17） | 0.99（0.78,1.26） |
| Age (years) | 18-44 | - | - | - | - | - | - | - | - | - |
|  | ≧45 | 1.03(0.81,1.31) | 0.94(0.78,1.13) | 1.04（0.87,1.24） | 0.85（0.64,1.12） | - | Chow text p=0.402 | 0.91（0.75,1.11） | 1.04（0.80,1.36） | 0.99(0.64,1.55) |
| bachelor degree | NO | - | - | - | - | - | ^ | - | - | - |
|  | yes | 0.93(0.79,1.08) | 1.01(0.88,1.15) | 0.96（0.85,1.07） | 1.04（0.83,1.31） | 0.97(0.87,1.08) | 1.04（0.76,1.41） | 0.90（0.78,1.05） | 1.02（0.87,1.21） | 1.09(0.85,1.39) |
| Household income level | Low | - | - | - | - | - | - | - | - | - |
|  | Medium | 0.90(0.75,1.08) | 1.06(0.90,1.24) | 0.95（0.83,1.08） | 1.11（0.84,1.48） | 0.98(0.86,1.11) | 0.97（0.68,1.38） | 1.04（0.88,1.23） | 0.96（0.79,1.16） | 0.88(0.64,1.22) |
|  | High | 1.44(1.21,1.72)*** | 1.11(0.96,1.28) | 1.19(1.06,1.35）*** | 1.39（1.08,1.79）** | 1.24(1.11,1.40)*** | 1.17（0.85,1.61） | 1.17（0.99,1.38）* | 1.28（1.07,1.52）*** | 1.24(0.95,1.63) |
| Self- rated health status | Fair or poor | - | - | - | - | - | - | - | - | - |
|  | good | 1.36(0.81,2.28) | 1.20(0.82,1.77) | 1.41(0.92,2.14） | 1.09（0.68,1.76） | 1.43(1.02,2.02)** | 0.79（0.38,1.66） | 1.07（0.67,1.73） | 1.10（0.68,1.79） | 2.63(1.25,5.53)** |
|  | very good | 1.85(1.13,3.02)** | 1.74(1.21,2.50)*** | 1.83(1.22,2.73)*** | 1.79（1.15,2.80）** | 1.94(1.41,2.68)*** | 1.16（0.56,2.40） | 1.53(0.97,2.40)* | 1.50(0.95,2.39)* | 3.52(1.80,6.91)*** |
| Chronic disease | No | - | - | - | - | - | - | - | - | - |
|  | Yes | 1.31(1.09,1.57*** | 1.07(0.91,1.26) | - | Chow text p=0.41 | 1.23(1.08,1.41)*** | 0.95（0.71,1.27） | 1.19（0.98,1.44)* | 1.11（0.91,1.35） | 1.21(0.91,1.59) |
| COVID-19 infection (participants or family member) | No | - | - | - | - | - | - | - | - | - |
|  | Yes | 2.59(1.98,3.40)*** | 1.85(1.40,2.45)*** | 2.42（1.91,3.08）*** | 1.84（1.31,2.59）*** | 2.19(1.79,2.66)*** | 3.84（1.61,9.13）*** | 2.31(1.60，3.33)*** | 1.55（1.11,2.19）** | 2.49(1.79,3.46)*** |
| Lost job due to COVID-19 | No | - | - | - | - | - | - | - | - | - |
|  | Yes | 1.41(1.19,1.67)*** | 1.30(1.12,1.51)*** | 1.27（1.12,1.44）*** | 1.65(1.29,2.11)*** | 1.33(1.18,1.50)*** | 1.40（0.98,2.00）* | 1.30（1.08,1.55）*** | 1.41（1.19,1.68)*** | 1.28(0.98,1.66)* |
| Food shortage | No | - | - | - | - | - | - | - | - | - |
|  | Yes | 1.58(1.32,1.90)*** | 1.34(1.14,1.57)*** | 1.35（1.17,1.54）*** | 1.90(1.47,2.47)*** | 1.52(1.34,1.72)*** | 0.99（0.67,1.46） | 1.38（1.13,1.68）*** | 1.24(1.02,1.49)** | 2.18(1.67,2.83)*** |
| Drug shortage | No | - | - | - | - | - | - | - | - | - |
|  | Yes | 1.59(1.33,1.89)*** | 1.30(1.11,1.52)*** | 1.40（1.23,1.60）*** | 1.46（1.13,1.90）*** | 1.48(1.30,1.67)*** | 1.14（0.79,1.64） | 1.19（0.99,1.43）* | 1.47(1.22,1.78)*** | 1.93(1.47,2.53)*** |
| Daily activities affected by COVID-19-related financial strain | No difficulty at all | - | - | - | - | - | - | - | - | - |
|  | Mild difficulties | 1.05(0.86,1.26) | 1.11(0.96,1.29) | 1.10（0.97，1.24） | 0.92（0.69,1.23） | 1.09(0.96,1.23） | 0.94（0.69,1.29） | 1.12（0.96,1.31） | 1.04(0.86,1.28) | 1.03(0.67,1.58) |
|  | Extreme difficulties | 1.34(1.05,1.69)** | 1.32（1.07,1.63）*** | 1.36（1.14,1.62）*** | 1.12（0.78,1.59） | 1.32（1.12,1.56）*** | 1.33（0.81,2.21） | 1.55（1.21,1.99）*** | 1.13(0.87,1.46) | 1.37(0.90,2.10) |
| Perceived risks of infection | Low | - | - | - | - | - | - | - |  | - |
|  | Medium | 0.96(0.76,1.22) | 0.93(0.76,1.12) | 0.91（0.78,1.08） | 1.02（0.73,1.42） | 0.96（0.82,1.12） | 0.83（0.45,1.19） | 1.02（0.82,1.27） | 0.92(0.72,1.18) | 0.77(0.52,1.15) |
|  | High | 0.98(0.80,1.19) | 0.83(0.70,0.98)** | 0.87（0.86,1.01）* | 0.90（0.67，1.20） | 0.88(0.77,1.01)* | 0.79（0.53,1.19） | 0.98（0.81,1.18） | 0.84(0.67,1.04) | 0.77(0.56,1.06) |

Note: OR : Odds Ratio , CI : confidence interval, ***p<0.01, **p<0.05,*p<0.1,

Table s4 The multiple regression models among age, chronic disease , infection risk level of living area subgroups. (Physical Activity)

| Variables | | **Physical Activity** | **Physical Activity** | **Physical Activity** | **Physical Activity** | **Physical Activity** | **Physical Activity** | **Physical Activity** | **Physical Activity** | **Physical Activity** |
| --- | --- | --- | --- | --- | --- | --- | --- | --- | --- | --- |
|  |  | Male  N=3694 | Female  N=4734 | Responded “yes” No-Chronic disease Groups N=6745 | Responded “yes” Chronic disease Groups N=1683 | Responded “yes” Age=18-44 N=7387 | Responded “yes” age≧45 N=1041 | Responded “yes” Regional Risk level= Low N=3787 | Responded “yes” Regional Risk level=Medium N=3065 | Responded “yes” Regional Risk level=High N=1576 |
|  |  | OR(95%CI) | OR(95%CI) | OR(95%CI) | OR(95%CI) | OR(95%CI) | OR(95%CI) | OR(95%CI) | OR(95%CI) | OR(95%CI) |
| Restricted health service utilization | No | - | - | - | - | - | - | - | - | - |
|  | Yes | 1.27(1.08,1.50)*** | 1.06(0.93,1.22) | 1.12(0.99,1.26)* | 1.26(0.98,1.60)* | 1.12（1.00,1.25）** | 1.22(0.91,1.63) | 1.02(0.87,1.19) | 1.17(0.99,1.39)* | 1.51（1.14,1.99）*** |
| Region | City | - |  | - | - | - | - | - | - | - |
|  | Rural | 1.03(0.86,1.24) | 0.94(0.82,1.09) | 1.03(0.91,1.17) | 0.83(0.64,1.08) | 1.00（0.89,1.13） | 0.89(0.64,1.24) | 1.08(0.91,1.27) | 0.88(0.73,1.05) | 0.95（0.70,1.30） |
|  | Town | 1.18(0.94,1.48) | 0.83(0.69,0.99)** | 0.96(0.82,1.11) | 0.99(0.71,1.38) | 0.95（0.82,1.10） | 1.12(0.71,1.77) | 1.13(0.91,1.40) | 0.83(0.67,1.03)* | 0.95（0.68,1.33） |
| Risk level of  living area | Low | - | - | - | - | - | - | low vs med + high Chow text p=0.024 | low vs med Chow text p=0.124 | low vs high Chow text p=0.012 |
|  | Medium | 1.02(0.86,1.20) | 0.94(0.73,0.96)** | 0.87(0.78,0.98)** | 1.09(0.85,1.40) | 0.88（0.78,0.98）** | 1.09(0.81,1.46) |  |  |  |
|  | High | 1.33(1.06,1.67)** | 1.05(0.86,1.29) | 1.20(1.01,1.43)** | 1.05(0.77,1.45) | 1.15（0.97,1.35）* | 1.02(0.64,1.64) |  |  |  |
| Married status | Unmarried / divorced / widowed | - | - | - | - | - | - | - | - | - |
|  | Married | 1.46(1.25,1.71)*** | 1.28(1.11,1.47)*** | 1.36(1.22,1.53)*** | 1.20(0.94,1.53) | 1.39（1.25,1.55）*** | 0.66(0.39,1.10) | 1.38(1.18,1.62)*** | 1.24(1.05,1.46)** | 1.50(1.19,2.01)*** |
| Sex | Female | - | Chow text p=0.016 | - | - | - | - | - | - | - |
|  | Male | - |  | 1.37(1.23,1.53)*** | 1.08(0.87,1.34) | 1.34 (1.21,1.48)*** | 1.23 (0.94,1.61) | 1.23（1.07,1.42）*** | 1.41(1.21,1.65)*** | 1.36(1.05,1.75)** |
| Age (years) | 18-44 | - | - | - | - | - | - | - | - | - |
|  | ≧45 | 0.79(0.63,1.00)* | 0.87(0.82,1.05) | 0.87(0.73,1.04) | 0.70(0.53,0.92)** | ^ | Chow text p=0.32 | 0.81(0.67,0.99)** | 0.94(0.73,1.22) | 0.77（0.49,1.20） |
| bachelor degree | NO | - | - | - | - | - | ^ | - | - | - |
|  | yes | 1.06(0.91,1.25) | 1.05(0.92,1.21) | 1.06(0.94,1.19) | 1.04(0.83,1.31) | 1.06（0.95,1.18） | 1.08(0.79,1.47) | 1.10(0.95,1.29) | 0.99(0.84,1.17) | 1.28（0.99,1.65）* |
| Household income level | Low | - | - | - | - | - | - | - | - | - |
|  | Medium | 1.34(1.12,1.61)*** | 1.41(1.20,1.65)*** | 1.38(1.21,1.58)*** | 1.44(1.08,1.92)** | 1.39（1.22,1.58）*** | 1.31(0.93,1.86) | 1.39(1.17,1.65)*** | 1.56(1.29,1.90)*** | 0.99（0.71,1.39） |
|  | High | 1.15(0.96,1.37) | 1.33(1.15,1.53)*** | 1.27(1.12,1.43)*** | 1.21(0.95,1.56)* | 1.26（1.12,1.42）*** | 1.20(0.88,1.64) | 1.27(1.08,1.51)*** | 1.32(1.10,1.58)*** | 1.06（0.80,1.40） |
| Self- rated health status | Fair or poor | - | - | - | - | - | - | - | - | - |
|  | good | 1.69(1.04,2.77)** | 1.70(1.16,2.51)*** | 2.25(1.46,3.46)*** | 1.41(0.89,2.21) | 1.65（1.18,2.30）*** | 1.87(0.85,4.13) | 1.64(1.01,2.66)** | 1.79(1.10,2.94)** | 1.71（0.86,3.40） |
|  | very good | 4.39(2.75,7.03)*** | 4.11(2.84,5.94)*** | 5.80(3.83,8.78)*** | 3.11(2.02,4.78)*** | 4.09（2.99,5.60)*** | 4.52(2.08,9.85)*** | 4.82(3.02,7.69)*** | 3.94(2.45,6.34)*** | 3.18（1.72,5.88）*** |
| Chronic disease | No | - | - | - | ^ | - | - | - | - | - |
|  | Yes | 0.83(0.69,1.00)** | 1.00(0.84,1.19) | Chow text p=0.224 | ^ | 0.94(0.81,1.08)* | 0.87(0.65,1.16) | 0.97(0.80,1.17) | 1.04(0.85,1.28) | 0.67（0.50,0.88）*** |
| COVID-19 infection (participants or family member) | No | - | - | - | - | - | - | - | - | - |
|  | Yes | 1.88(1.40,2.53)*** | 1.51(1.11,2.04)*** | 1.76(1.34,2.31)*** | 1.76(1.24,2.48)*** | 1.58(1.27,1.96)*** | 4.35(1.65,11.48)*** | 1.90(1.26,2.85)*** | 1.23(0.86,1.79) | 1.87（1.31,2.66）*** |
| Lost job due to COVID-19 | No | - | - | - | - | - | - | - | - | - |
|  | Yes | 1.02(0.85,1.21) | 1.20(1.03,1.40)** | 1.04(0.92,1.19) | 1.40(1.09,1.80)*** | 1.15(1.01,1.30)** | 0.89(0.62,1.28) | 1.05(0.87,1.26) | 1.06(0.89,1.27) | 1.33（1.01,1.74）*** |
| Food shortage | No | - | - | - | - | - | - | - | - | - |
|  | Yes | 1.49(1.23,1.82)*** | 1.12(0.95,1.32) | 1.21(1.05,1.40)*** | 1.50(1.15,1.96)*** | 1.38(1.20,1.57)*** | 0.61(0.41,0.90)** | 1.16(0.94,1.42) | 1.15(0.95,1.40) | 1.72（1.29,2.29）*** |
| Drug shortage | No | - | - | - | - | - | - | - | - | - |
|  | Yes | 1.27(1.05,1.54)** | 1.46(1.24,1.72)*** | 1.33(1.15,1.53)*** | 1.51(1.16,1.97)*** | 1.40(1.23,1.60)*** | 1.17(0.81,1.69) | 1.09(0.90,1.33) | 1.61(1.32,1.96)*** | 1.49（1.11,2.00）*** |
| Daily activities affected by COVID-19-related financial strain | No difficulty at all | - | - | - | - | - | - | - | - | - |
|  | Mild difficulties | 0.93(0.77,1.12) | 0.92(0.80,1.07) | 0.98(0.86,1.11) | 0.74(0.56,0.98)** | 0.93(0.82,1.05) | 0.95(0.70,1.29) | 0.94(0.81,1.10) | 1.07(0.89,1.30) | 0.75（0.49,1.16） |
|  | Extreme difficulties | 0.91(0.71,1.16) | 1.10(0.86,1.36) | 1.12(0.94,1.35) | 0.64(0.45,0.91)** | 0.99(0.84,1.17) | 1.20(0.72,2.00) | 1.07(0.83,1.38) | 1.06(0.82,1.37) | 0.90（0.58,1.39） |
| Perceived risks of infection | Low | - | - | - | - | - | - | - | - | - |
|  | Medium | 1.24(0.98,1.58)* | 0.86(0.70,1.05) | 0.89(0.75,1.06) | 1.54(1.11,2.14）** | 1.04(0.88,1.22) | 0.67(0.41,1.09) | 0.92(0.74,1.15) | 1.06(0.82,1.37) | 1.16（0.77,1.74） |
|  | High | 1.08(0.89,1.31) | 0.71(0.59,0.84)*** | 0.94(0.63,0.85)*** | 1.51（1.14,2.00）*** | 0.83(0.73,0.96)** | 0.87(0.58,1.31) | 0.83(0.69,0.99)** | 0.84(0.67,1.05) | 1.06（0.76,1.46） |

Note: OR : Odds Ratio , CI : confidence interval, ***p<0.01, **p<0.05,*p<0.1,
